# Decrypting the Origin and Pathogenesis in Pregnant Ewes of a New Ovine Pestivirus Closely Related to Classical Swine Fever Virus

**DOI:** 10.3390/v12070775

**Published:** 2020-07-17

**Authors:** Miaomiao Wang, Enrica Sozzi, José Alejandro Bohórquez, Mònica Alberch, Joan Pujols, Guillermo Cantero, Alessandra Gaffuri, Davide Lelli, Rosa Rosell, Albert Bensaid, Mariano Domingo, Lester Josue Pérez, Ana Moreno, Llilianne Ganges

**Affiliations:** 1 OIE Reference Laboratory for Classical Swine Fever, IRTA-CReSA, 08193 Barcelona, Spain; miaomiao.wang@irta.cat (M.W.); josealejandro.bohorquez@irta.cat (J.A.B.); monica.alberch@irta.cat (M.A.); joan.pujols@irta.cat (J.P.); guillermo.cantero@irta.cat (G.C.); rosa.rosell@irta.cat (R.R.); albert.bensaid@irta.cat (A.B.); Mariano.Domingo@uab.cat (M.D.); 2 Istituto Zooprofilattico Sperimentale della Lombardia e Dell’Emilia Romagna, Via Antonio Bianchi 7/9, 25124 Brescia, Italy; enrica.sozzi@izsler.it (E.S.); alessandra.gaffuri@izsler.it (A.G.); davide.lelli@izsler.it (D.L.); anamaria.morenomartin@izsler.it (A.M.); 3 Departament d’Agricultura, Ramadería, Pesca i Alimentació (DARP), 08007 Generalitat de Catalunya, Spain; 4 Servei de Diagnòstic de Patologia Veterinària (SDPV), Departament de Sanitat I d’Anatomia Animals, Universitat Autònoma de Barcelona, Bellaterra, 08193 Barcelona, Spain; 5 Department of Clinical Veterinary Medicine, College of Veterinary Medicine, University of Illinois at Urbana-Champaign, Urbana, IL 61802, USA; lesterjosue@gmail.com

**Keywords:** pestivirus, OVPV, CSFV, cross-reactivity, trans-placental transmission, congenital persistent infection, phylodynamic, evolution

## Abstract

This study shows the origin and the pathogenic role of a novel ovine pestivirus (OVPV) isolated in 2017 in Italy, as a pathogenic agent causing severe abortions after infection in pregnant ewes and high capacity for virus trans-placental transmission as well as the birth of lambs suffering OVPV-persistent infection. The OVPV infection induced early antibody response detected by the specific ELISA against classical swine fever virus (CSFV), another important virus affecting swine. The neutralizing antibody response were similar against CSFV strains from genotype 2 and the OVPV. These viruses showed high identity in the B/C domain of the E2-glycoprotein. Close molecular diagnostics cross-reactivity between CSFV and OVPV was found and a new OVPV molecular assay was developed. The phylodynamic analysis showed that CSFV seems to have emerged as the result of an inter-species jump of Tunisian sheep virus (TSV) from sheep to pigs. The OVPV and the CSFV share the TSV as a common ancestor, emerging around 300 years ago. This suggests that the differentiation of TSV into two dangerous new viruses for animal health (CSFV and OVPV) was likely favored by human intervention for the close housing of multiple species for intensive livestock production.

## 1. Introduction

The *Pestivirus* genus, belonging to the Flaviviridae family, is one of the most relevant in animal health. Pestiviruses are distributed worldwide, being responsible for generating a variety of economically-important diseases in domestic and wildlife animals including ruminants and swine [1]. The best-known species are bovine viral diarrhea virus 1 (BVDV-1), bovine viral diarrhea virus 2 (BVDV-2), classical swine fever virus (CSFV), and border disease virus (BDV), classified as *Pestivirus A, B, C,* and *D*, respectively. Recently, seven new species have been added to this genus, named from *E* to *K*, including the atypical porcine pestivirus (APPV) which generates congenital tremors in piglets [2,3,4,5]. Moreover, the lateral-shaking-inducing neurodegenerative agent (LINDA) has been reported as a new *Pestivirus* different from the other eleven mentioned above, constituting number 12 of this growing list of viruses [6].

The *Pestivirus* genome consists of a single plus-stranded RNA, which contains one large open reading frame (ORF) flanked by two untranslated regions (UTRs). The ORF encodes a polyprotein of approximately 3900 amino acids, which is subsequently processed by cellular and viral proteases into mature proteins—four structural proteins (C, Erns, E1, and E2) and eight non-structural proteins (Npro, P7, NS2, NS3, NS4A, NS4B, NS5A, and NS5B) [7].

*Pestivirus* infections may be subclinical or produce a range of clinical conditions characterized by acute diarrhea, acute hemorrhagic syndrome, acute fatal disease, and wasting disease. Pestiviruses have the ability to generate congenital infections by trans-placental transmission that can result in fetal death, congenital abnormalities, or animals born with persistent lifelong infection [8]. Animals with persistent infection play an important role in the epidemiology of pestiviruses in swine and ruminants. The host range is variable depending on the *Pestivirus* species; some pestiviruses such as BVD-1, BVD-2, and BDV, with ruminants as main hosts, are able to cross species barriers and infect a wide range of hosts [9,10]. By contrast, others like CSFV have a restricted natural host range and infect only swine including wild and domestic pigs. CSFV is the causative agent of classical swine fever (CSF), a highly contagious viral disease that causes devastating epidemics. The disease is notifiable to the World Organisation for Animal Health (OIE) due to its huge economic impact. CSF is still endemic in some regions of Asia and Central and South America [11].

In 2017, a novel *Ovine pestivirus* (OVPV) was isolated from aborted lamb fetuses in North Italy. The analysis of the complete sequences of the OVPV isolates showed a high percentage of identity and formed a well-supported single clade distinct from other known pestiviruses, although it was related to the CSFV clade [12]. In addition, the new OVPV showed a higher sequence identity to CSFV across the whole genome (72.2%) than with other *Pestivirus* sequences isolated from sheep. Sequence identity between OVPV and CSFV was as high as 89.9% in the 5′UTR region. These results revealed that the Italian OVPV is more closely related to CSFV than BDV, BVDV, or any other of the existing or recently-discovered pestiviruses, such as Aydin, LINDA, or APPV [12,13,14].

The present work focused on reproducing, for the first time, in an experimental infection, the capacity of OVPV to infect pregnant sheep, in order to fulfil Koch’s postulates and to study the kinetics of viral replication and pathogenesis. The virus generated reproductive failure, such as abortion, with vertical transmission and congenital persistent infection. To get a better understanding of the OVPV origin and its relationship with CSFV and other members of *Pestivirus* genus with their respective vertebrate hosts, a co-evolutionary analysis was also performed. In addition, in the present study, cross-reactivity with CSFV in the molecular diagnosis was also evaluated.

## 2. Materials and Methods

### 2.1. Cells and Viruses

The porcine kidney cell line PK-15 ATCC (CCL-33) and the MDBK ATCC (CCL-22) cells were obtained from the ATCC. The fetal sheep thymus cell line (SFT-R) was obtained from the Cell Culture Collection of Veterinary Medicine, Friedrich-Loeffler Institute, Island of Riems, Germany. The three cell lines were tested as *Pestivirus*-free, and the PK-15, MDBK and SFT-R cells were grown in Eagle’s minimum essential medium supplemented with 5% fetal calf serum. The viruses were titrated in MDBK and SFT-R cells in 96-well tissue culture plates. Viral replication was monitored by immune peroxidase monolayer assay (IPMA) with a swine polyclonal *Pestivirus* antibody [15]. Viral titers were determined by endpoint dilution and the 50% tissue culture infective dose (TCID_50_) per milliliter was calculated using standard statistical methods [16].

The Italy OVPV, recently isolated, was used for in vivo assays [12]. The CSFV strain, Alfort/187, the CSFV Diepholz1/Han94 strains, and the BVDV NADL strain were kindly provided by the CSFV EU Reference Laboratory (EURL), Hannover, Germany. The BDV 137/4 was kindly provided by the Central Veterinary Laboratory (CVL), Weybridge, UK and the BDV-pig-SP-2007 isolated in Spain was also used [17]. The CSFV Margarita and Catalonia01 strains were also employed in the neutralization assays [18].

### 2.2. Experimental Infection in Pregnant Ewes

Eight ewes, *Pestivirus*-free at 63 days of gestation (dg) were allocated in one box from the Biosafety Level 3 (BSL3) in the animal facilities in CReSA, Barcelona, Spain. The origin farm was reported to be free of *Chlamydophila abortus* and Q fever. Before entering into the BSL3 facility, the ewes were administered two intramuscular doses of oxytetracycline (20 mg/kg), one at 24 h before transport and the other at the moment of arrival at CReSA. After five days of acclimation period (68 dg) and according to previously-described protocols for experimental infections with pestiviruses in ruminants [19,20], the eight animals were intramuscularly injected into the hind leg muscles with 5 × 10^6^ TCID_50_ of a OVPV viral isolate [12]. A daily clinical evaluation was carried out and the clinical signs were blindly registered by veterinary staff throughout the trial. Serum, whole blood, and nasal and rectal swab samples were collected at the day of inoculation, and every week after that, until the end of the study, 10 days after the date of delivery. However, in the days near to delivery, the sample collection was performed taking into account the clinical status. After delivery, lamb samples were also collected in the first week after lambing.

Animals that aborted were euthanized and placenta, tonsil, brain, lung, liver, spleen, and kidney tissues were collected. Likewise, tissue samples (tonsil, thymus, brain, lung, liver, spleen, and kidney) were collected from fetuses or lambs in the case of abortion, stillbirth, or death. The same samples were collected from animals that carried the pregnancy to term and their lambs, after euthanasia at 10 days after delivery (end of the experiment). Blood samples for sera collection and nasal and rectal swabs were also collected from the stillbirths when possible. The experiment was performed in accordance with European regulations and approval by the Ethical Committee of the Generalitat de Catalonia, Spain, under the animal experimentation project number 10631 of the 23^rd^ of January 2020.

### 2.3. Pestivirus and CSFV-Specific RNA Detection

Tissue samples were homogenized in Eagles sterile Minimal Essential Medium (EMEM; 1 g of tissue + 9 mL EMEM) 6% supplemented with penicillin 10,000 Units/mL, streptomycin 10,000 U/mL, and nystatin antibiotics 10,000 U/mL, and tested for *Pestivirus* RNA detection. RNA was extracted using the MagAttract 96 cador Pathogen Kit (Qiagen), following the manufacturer’s instructions. In all cases, extraction was performed from an initial sample volume of 200 μL to obtain a final volume of 100 μL of RNA, which was stored at −80 °C. The presence of *Pestivirus* RNA in the blood, serum, tissues, and in nasal and rectal swabs was analyzed by the quantitative reverse transcription-PCR (RT-qPCR) [21] and CSFV-specific RT-qPCR [22]. Threshold cycle (*C*t) values equal to or lower than 40 were considered as positive. Samples in which fluorescence was undetectable were considered as negative. As previously described, *C*t values from 10 to 23 were considered as high, from 23 to 29 as moderate, and between 29 and 42 as low RNA viral load [23]. Positive RT-PCR samples in tissue samples were inoculated into cell cultures for virus isolation in SFT-R cells by peroxidase linked assay (PLA) test.

### 2.4. Design and Validation of a New RT-qPCR for the Specific Detection of OVPV RNA

Two primers and one probe were designed for specific detection of the OVPV sequence by targeting the NS5B gene, as follows: forward primer (10236–10257), 5′-CAAGGTGGCTGAGCAATTACAG-3′; reverse primer (10335–10356), 5′-CTGAGCTCTCACTACAGGCAGT-3′, and probe (10284–10305), 5′-CAATAAAGTAATAGGCTCGACC-3′. The nucleotide positions were based on the genome sequence of the OVPV strain (GenBank accession number MG770617.1). The probe was labeled with 6-FAM at the 5´ end, using a TAMRA quencher at the 3´end. The primers and probe were purified by reverse phase HPLC. The one-step RT-qPCR protocol was undertaken using the commercially available AgPath-ID™ One-Step RT-PCR Reagents kit (Applied Biosystems ThermoFisher). The RT-qPCR assay was optimized using a total volume of 25 μL and was performed using an Applied Biosystems^®^ 7500 Fast Real-Time PCR System. Cycling steps for the assay included a reverse transcription at 48 °C for 10 min, activation of DNA polymerase at 95 °C for 10 min, and 40 cycles of 97 °C for 2 s and 61 °C for 30 s. To test specificity and sensitivity of the newly-developed assay, an RNA panel was prepared including other 23 samples from the *Pestivirus* reference strains (Table 1).

### 2.5. Antibody Responses Characterization after OVPV Infection

To determine antibodies against ruminant pestiviruses, the BVDV/MD/BDV p80 Protein Antibody Competitive ELISA Test was used following the manufacturer’s instructions (IDEXX Laboratories, Montpellier, France). Results are determined by the sample absorbance at 450 nm (A_450_)/negative control mean percentage (S/N%). Values above 50% were considered as negative, between 40% and 50% were considered doubtful and S/N% values lower than or equal to 40% were considered as positive. In addition, CSFV E2-specific antibodies after infection were detected by ELISA using the commercial CSFV Ab test (IDEXX Laboratories, Liebfeld, Switzerland) according to the manufacturer’s recommendations. The samples were considered positive when the blocking percentage was ≥40%.

In order to evaluate the neutralizing antibody response after the infection, sera samples were also tested by neutralization peroxidase-linked assay (NPLA) [26] against the homologous strain (OVPV) and heterologous *Pestivirus* strains, CSFV (Alfort/187, Margarita, Catalonia01, and Diepholz1/Han94 strains), BVDV (NADL strain), and BDV (137/4 and BDV-pig-SP-2007 strains). The titers were expressed as the reciprocal dilution of serum that neutralized 100 TCID_50_ in 50% of the culture replicates [16].

To further characterize the antigenic relationship of the novel OVPV and other well-known *Pestivirus* members the mean of neutralizing titers obtained were compared by Brown–Forsythe Welch ANOVA test with multiple comparisons using Dunnett correction all implemented in GraphPad Prims Software (GraphPad software, Inc. 1992-2020). In addition, the sequence identities of the B/C domain, within the E2 glycoprotein, of CSFV (Alfort/187 and Diepholz1/Han94 strain), BVDV (NADL strain), BDV (137/4 and BDV-pig-SP-2007 strains), and Tunisian sheep virus (TSV), were analyzed applying the identity matrix distance computed in the BioEdit software package.

### 2.6. Evolutionary Time Reconstruction and Cophylogenetic Analysis

The evolutionary history of the *Pestivirus* genus members and their vertebrate hosts were estimated following a previously-reported methodology [27]. Briefly, sequences from the E2 gene of all *Pestivirus* members reported (Table S1) and cyt b sequences for their vertebrate hosts (Table S2) were used to generate the BEAST input file by BEAUti within the BEAST package [28] v1.10.434 (freely available at http://beast.bio.ed.ac.uk). The time for the most recent common ancestor (tMRCA) were estimated employing a Bayesian Markov chain Monte Carlo (MCMC) approach. The model selection was performed as described in a previous study [27]. MCMC chains were run for 2 × 10^8^ generations, in order to obtain an ESS > 250, and the first 10% trees were discarded as ‘‘burn-in’’. Convergence was assessed by estimating the effective sampling size (ESS) after a 10% burn-in, using Tracer software version 1.5 (http://tree.bio.ed.ac.uk/software/tracer/). The trees with maximum log clade credibility were selected and visualized by FigTree v1.1.2 [29]. The presence of cophylogeny, the degree of co-speciation between *Pestivirus* species and their vertebrate hosts, was tested using A Novel Procrustes Application to Cophylogenetic Analysis (PACo) [30]. In addition, a tree-reconciliation using Jane software v4 [31] was evaluated. Jane software assigns costs to four evolutionary events—cospeciation, duplication, host switch, and sorting. Additionally, a cost to failure to divergence was assigned [31]. Thus, cost was set to obtained minimal values describing the tree-reconciliation events.

## 3. Results

### 3.1. Specific and Sensitive Detection of OVPV RNA by the Newly-Designed Real Time TaqMan Assay

A new RT-qPCR assay was designed for the specific detection of the new OVPV RNA. The new assay was validated using RNA samples from other 23 *Pestivirus* reference strains found in Table 1. Positive results were considered for Ct values equal to or less than 40. This RT-qPCR was found to only detect the new OVPV. The sensitivity of the assay was estimated to be as high as 0.4 TCID. The assay had a reaction coefficient (R2) of 0.994.

### 3.2. OVPV Causes a High Proportion of Abortions and Stillbirths in Pregnant Ewes after Infection

**Figure 1:**
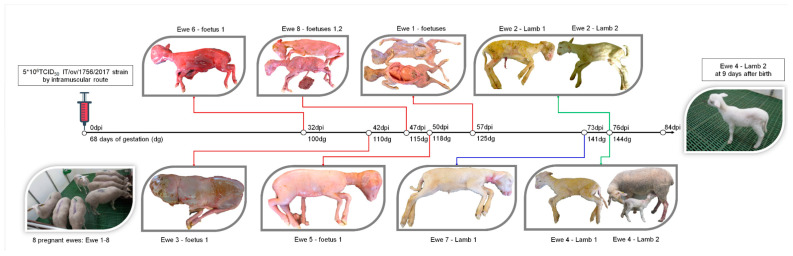
Evolutionary timeline of abortions and stillbirths after *Ovine pestivirus* (OVPV) infection in pregnant ewes. Pregnant ewes 1 to 8 were infected with OVPV at 68 days of gestation. The ewes were monitored clinically on a daily basis during the 84 days of the study. The abortions (red line), premature stillbirth (blue line), and non-premature stillbirth (green line) of ewes are shown. Lambs that reached term and were born alive are also shown.

Clinical signs were monitored during the 84 days of the trial. No systemic clinical signs were observed in the eight pregnant ewes. However, five out of eight ewes (62.5%, numbers 1, 3, 5, 6, and 8) aborted between the second and last third of gestation, ranging from 100 to 125 dg or 32 to 57 days post-infection (dpi) (Figure 1). The seven fetuses that were aborted by the five ewes were found to be either mummified or showing signs of reabsorption. Furthermore, organs were poorly defined, showing signs of autolysis (Figure 1).

One of the three ewes that did not abort, animal 7, gave premature birth to one lamb at 141 dg (73 dpi). This animal was unable to stand up and feed due to severe weakness and was euthanized on the day of birth. On the other hand, the last two ewes to give birth, did so at a normal lambing time (144 dg). Ewe 2 gave birth to two stillbirths, whereas ewe 4 gave birth to one stillbirth and one live lamb. The live lamb showed hairy fleece and moderate weakness but was still able to feed from the ewe (Figure 1). However, from day 2 on, this animal developed bilateral conjunctivitis and diarrhea. Afterwards, an increase in body temperature (40.4 °C), accompanied by tremor and polyarthritis were registered, during the seven days of life of this animal.

### 3.3. Viral Replication Capacity and Shedding of OVPV in the Infected Ewes

In order to detect the capacity of the virus to induce viremia, as well as its excretion during infection, two RT-qPCR assays were performed. One of them had been previously developed for *Pestivirus* detection [21] and the other was the novel RT-qPCR assay, designed in the present study for the specific detection of the novel OVPV.

**Figure 2:**
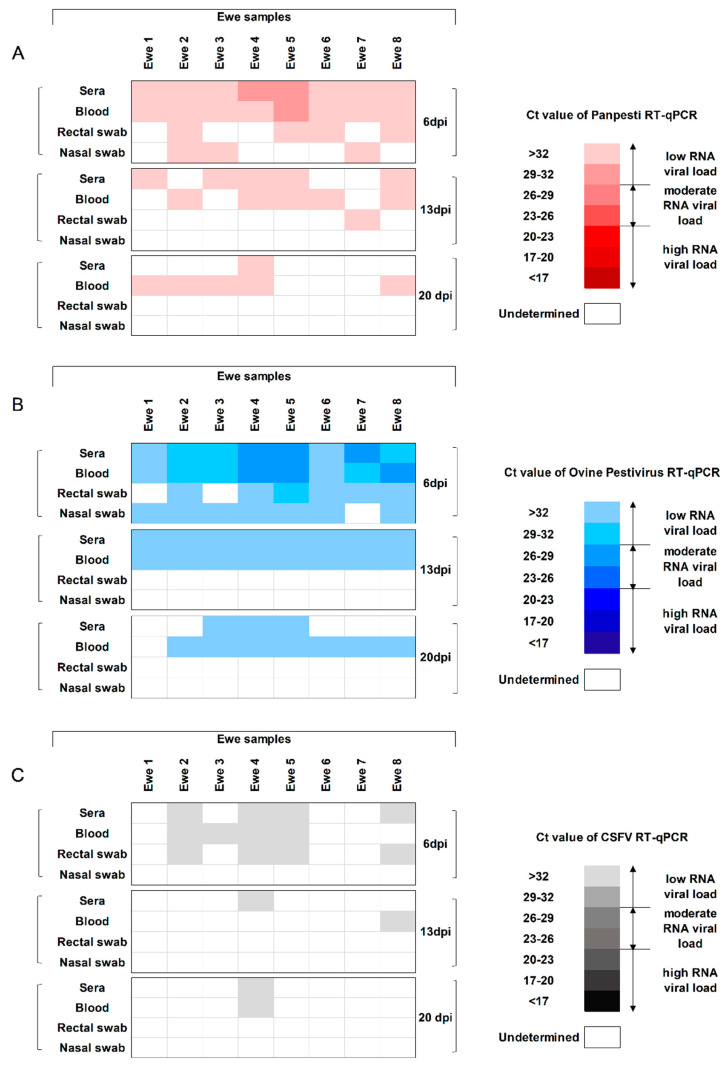
Weekly detection of OVPV RNA in sera, blood, and nasal and rectal swabs from infected ewes. The OVPV RNA was analyzed by (**A**) *Pestivirus* RT-qPCR [21], (**B**) the new specific OVPV RT-qPCR, and (**C**) specific CSFV RT-qPCR [22]. The RNA load, according to the Ct value, is represented as low, moderate, or high by the intensity of the red, blue, or gray colors in the scale for (**A**–**C**), respectively. Ct values above 40 were considered as negative.

OVPV RNA was detected in sera and blood from all the ewes at 6 dpi by both RT-qPCR assays, with viral RNA load ranging from low to moderate (Figure 2A,B). At 13 dpi, all the ewes showed low RNA load by the specific OVPV RT-qPCR in both sera and blood. Meanwhile, three out of eight ewes were negative by the *Pestivirus* detection assay in each of these samples. The Ct values for the positive samples in both RT-qPCR assays corresponded with low RNA load (Figure 2A,B). Afterwards, viral RNA was found in seven blood samples by the specific OVPV RT-qPCR at 20 dpi, whereas it was only detected in three sera samples at this date. A low RNA load was found in all positive samples. At this time-point, lower detection capacity was found for the Panpesti RT-qPCR assay, with viral RNA being detected in one serum and five blood samples, always with a low viral RNA load (Figure 2A,B). From 27 dpi and until the end of the trial, OVPV RNA was sporadically detected only in blood samples, always with a higher proportion of positive animals for the specific OVPV RT-qPCR. Ct values corresponded with a low RNA load.

The excretion of OVPV was found during a short time after infection. The majority of OVPV RNA-positive nasal and rectal swab samples being found at 6 dpi by both RT-qPCR techniques and only one rectal swab sample was positive at 13 dpi by the *Pestivirus* RT-qPCR. From 20 dpi and until the end of the trial, absence of excretion capacity was found, as all the rectal and nasal swab samples were negative (Figure 2A,B).

**Figure 3:**
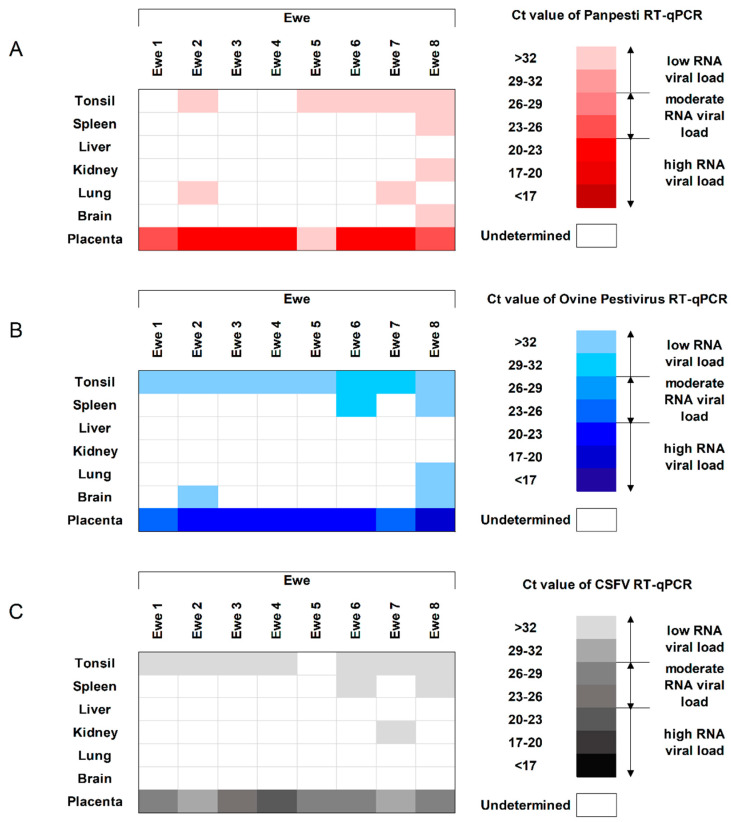
Detection of OVPV RNA in ewe tissues. The OVPV RNA analyzed by (**A**) *Pestivirus* RT-qPCR [21], (**B**) the new specific OVPV RT-qPCR, and (**C**) specific CSFV RT-qPCR [22]. The RNA load, according with the Ct value, is represented as low, moderate, or high by the intensity of the red, blue and gray colors in the scale for (**A**–**C**), respectively. Ct values above 40 were considered as negative.

Finally, to evaluate the replication capacity of OVPV in different tissues from the infected animals, tonsil, spleen, liver, kidney, lung, brain, and placenta were analyzed. Tonsil and placenta were identified as the main targets for replication. Even though the viral RNA load was low for both RT-qPCR assays, the specific OVPV RT-qPCR detected higher OVPV RNA load in all the ewes (Figure 3A,B). Notably, all of the placenta samples were positive and most of them showed a high OVPV RNA load by both molecular assays. However, the specific OVPV RT-qPCR assay detected higher viral RNA load than the Panpesti assay (Figure 3A,B).

### 3.4. Efficient Vertical Transmission Capability and Generation of OVPV Congenitally-Persistently-Infected Lambs

**Figure 4:**
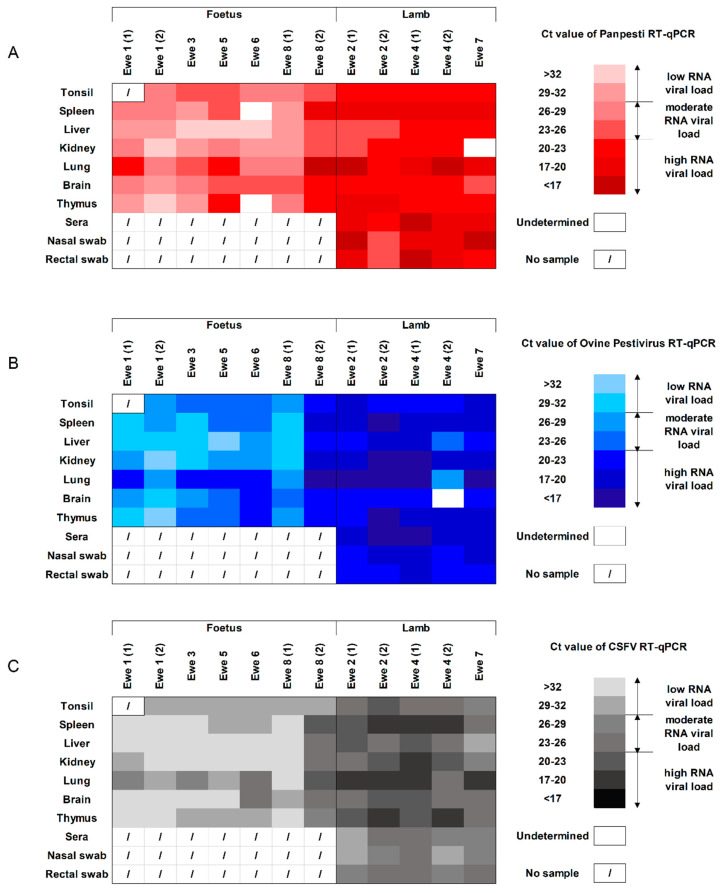
Detection of OVPV RNA in samples and tissues from foetuses and lambs. The OVPV RNA analyzed by **(A**) *Pestivirus* RT-qPCR [21], (**B**) the new specific OVPV RT-qPCR, and (**C**) specific CSFV RT-qPCR [22]. The RNA load, according with the Ct value, is represented as low, moderate, or high by the intensity of the red, blue and gray colors in the scale for (**A**–**C**), respectively. Ct values above 40 were considered as negative.

To evaluate the trans-placental transmission capacity of OVPV, tissues were collected from all the aborted fetuses. All the tissue samples from fetuses were positive for OVPV RNA by both RT-qPCR assays, showing mostly moderate or high RNA loads. Notably, the lung sample was found to have the highest OVPV RNA load from all the tissues analyzed (Figure 4A,B).

In addition, to assess the congenital infection capacity of OVPV, sera, tissues, and nasal and rectal swabs from stillbirths and live lambs were also analyzed. All the analyzed samples from lambs and stillbirths showed between moderate and high OVPV RNA load (Figure 4A,B). The highest RNA load was found in sera samples, as well as lungs, kidneys, thymus, and tonsils. These results were validated by viral isolation test in the lung samples, with viral titers around 10^6^ TCID_50_.

### 3.5. The OVPV Viral Replication in Ewes, Fetuses, and Lambs Cross-Reacts with the Molecular Diagnosis of CSFV

In order to determine the capacity for cross-reaction of the OVPV replication with the molecular diagnosis of CSFV, the specific RT-qPCR for CSFV RNA detection [22] was also performed. The replication and excretion of OVPV was found to cross-react with the CSFV-specific RT-qPCR assay at 6 dpi in half of the infected animals, although showing low RNA load. Afterwards, at 13 and 20 dpi, some sera and blood samples were positive by the CSFV RT-qPCR (Figure 2).

In the ewe tissues, in accordance with the previously performed molecular assays, the CSFV-specific RT-qPCR was also positive in all the placenta and most of the tonsil samples analyzed, highlighting the moderate to high RNA load found in placenta (Figure 3). Similar profile and viral replication level were found between the two previously performed RT-qPCR tests and the CSFV-specific molecular assay in the samples from fetuses, lambs and stillbirths (Figure 4).

### 3.6. The OVPV Induced High and Early CSFV-Specific Antibody Response Detected by ELISA

**Figure 5:**
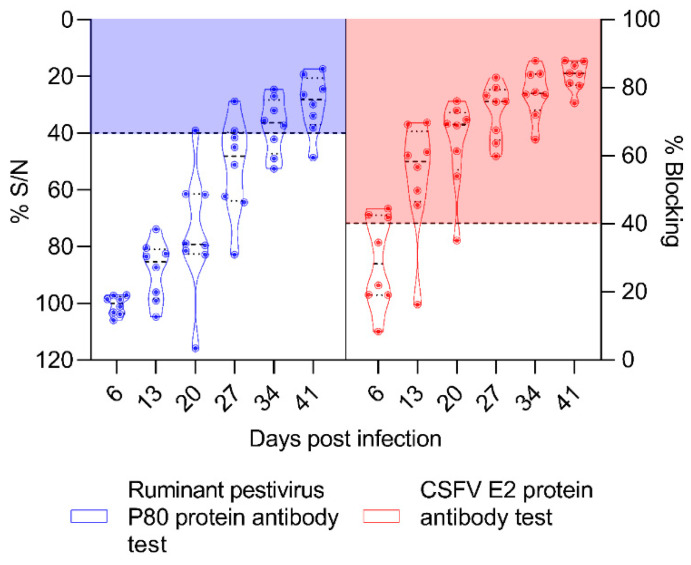
Kinetic of the humoral immune response induced in pregnant ewes after OVPV infection. The antibody responses against ruminant *Pestivirus* P80 protein (blue panel) and CSFV E2 glycoprotein (red panel) was monitored by ELISA test during the six weeks after OVPV infection. The anti-P80 antibody response is represented as sample A_450_/negative control mean percentage (% S/N, left γ-axis) with values over 50% being considered as negative, between 40–50% as doubtful, and under or equal to 40% as positive. The anti-E2 antibody response is represented as blocking percentage (% blocking, right γ-axis), with values under 30% being considered as negative, between 30–40% as doubtful, and over 40% as positive.

To assess the antibody response induced in ewes after OVPV infection, two ELISA tests were performed, one specific for detection of antibodies against ruminant pestiviruses and another for the evaluation of CSFV-specific anti-E2 antibodies. The majority of animals were positive at 34 and 41 dpi for ruminant pestivirus antibodies. Meanwhile, CSFV-specific antibody response was detected from 6 dpi and increased between 13 and 41 dpi, reaching a mean blocking percentage of 83% for all ewes at this time (Figure 5).

### 3.7. OVPV Activates an Efficient Neutralizing Antibody Response that Cross-Reacts with CSFV and Other Pestiviruses

To assess the neutralizing antibody response in the infected ewes, neutralizing antibody titers against OVPV were evaluated weekly in sera from these animals during the first six weeks of the trial by NPLA [26]. Moreover, the cross-reactivity of the neutralizing antibodies was also analyzed at 41 dpi against representative CSFV strains from genotypes 1 and 2, as well as other strains from different pestiviruses, such as BVDV and BDV. Neutralizing antibody response against OVPV was detected in all the pregnant ewes starting at the second week post-infection (13 dpi), ranging from 1/80 to 1/640, with a mean value of 1/290 (Table 2). This response increased in all the ewes throughout the experiment, reaching titers between 1/120 and 1/10240 at 41 dpi (mean value 1/4480). Cross reactivity was found for BVDV, ruminant BDV, and porcine BDV, with the mean neutralizing antibody titers of 1/64, 1/168, and 1/185, respectively (Table 2). Notably, two different profiles were observed in the analysis with the different CSFV strains. On the one hand, the cross-reactivity against CSFV Alfort/187 or Margarita strain (CSFV strains from genotype 1) was found to be the lowest of the pestiviruses analyzed, with a mean value between 1/44 and 1/51. Meanwhile, the highest cross-reaction for the neutralizing antibodies was detected against the CSFV Diepholz1/Han94 and CSFV Catalonia01 strain (CSFV from genotype 2), with titers similar or even higher than those detected against the OVPV homologous strain, ranging from 1/320 to 1/30720 (mean value between1/1960 and 1/7555) (Table 2).

**Figure 6:**
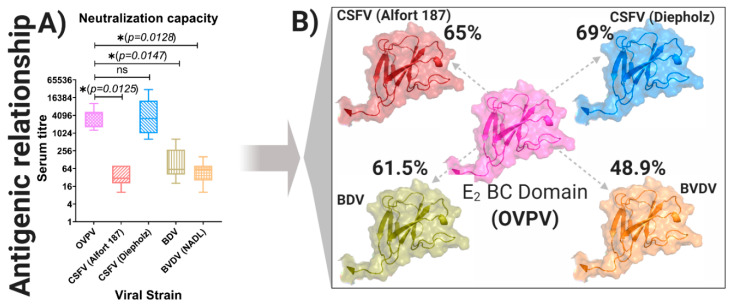
Antigenic relationship among the novel OVPV and other *Pestivirus* members. (**A**) Neutralization capacity deduced from the serum–antigen interactions (see Table 2). Mean values were compared by Brown–Forsythe Welch ANOVA test with multiple comparisons using Dunnett correction all implemented in GraphPad Prims Software (GraphPad software, Inc. 1992–2020). Abbreviation for each *Pestivirus* species tested is denoted, asterisk indicates significant difference (*p* < 0.05), *p*-value is also shown. (**B**) Graphical representation of the amino acid sequence identity for the most immunogenic domain (BC) of the most immunogenic protein of the *Pestivirus* members (E2) among the ovine *Pestivirus* and other member tested in the viral neutralization assay. The BC domain for CSFV Alfort/187 and Diepholtz strains is denoted in blue, in red is denoted the BC domain of the *Pestivirus* species, BDV and BVDV, the BC domain of the OVPV is denoted in pink. The percentage value for the identity of sequence is also shown.

The antigenic relationship of the OVPV was significantly different with the CSFV Alfort/187 strain (*p* = 0.0125), the BVDV, NADL strain (*p* = 0.0128), and the BDV-pig-SP-2007 strain (*p* = 0.0128) (Figure 6A). Interestingly, no difference was found regarding the antigenic relationship of the OVPV with the CSFV Diepholz1/Han94 strain. The sequence identities of the B/C domain of the E2 glycoprotein, the most immunogenic protein of the pestiviruses E2 were analyzed and determined. Notably, the Diepholz1/Han94 strain showed the highest sequence identities of B/C domain with the OVPV (69%), being 65%, 61.5%, and 48.9% for the CSFV Alfort/187 strain, BDV-pig-SP-2007 strain, and BVDV NADL strain, respectively (Figure 6B).

### 3.8. Evolutionary History and Cophylogenetic Reconstruction of OVPV and Their Vertebrate Hosts

**Figure 7:**
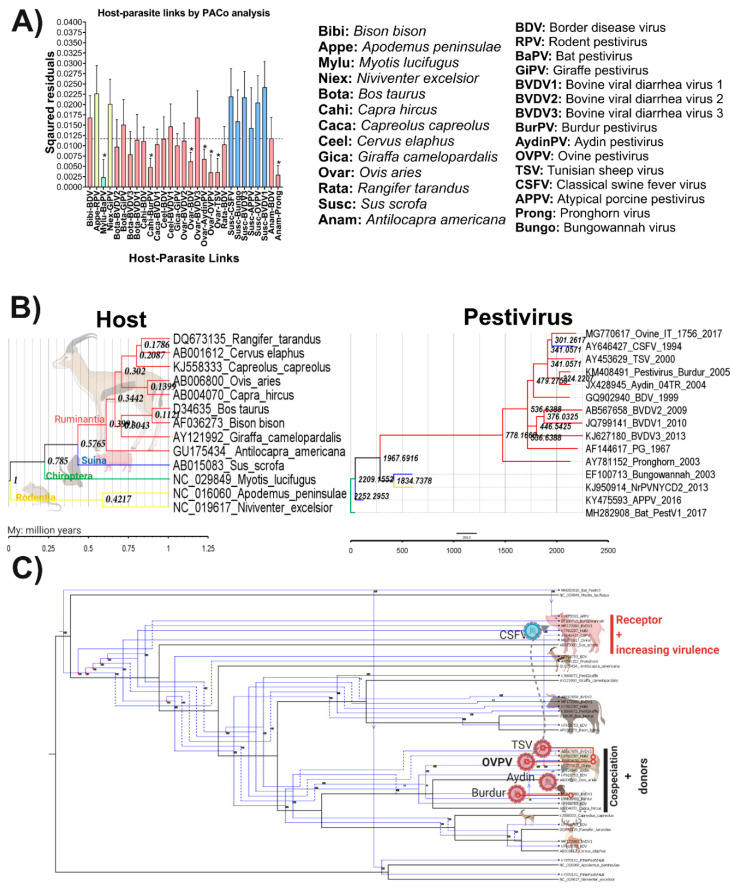
Viral–host coevolutionary history reconstruction for the *Pestivirus* genus. (**A**) Contributions of individual host–parasite links to the procrustean fit (right)—jacknifed squared residuals (bars) and upper 95% confidence intervals (error bars) resulting from applying PACo to patristic. Links supported among the *Pestivirus* species and their respective hosts were denoted (*). The median squared residual value obtained is represented by a dashed line. A pattern of color was used to facilitate the identification of the hosts—ruminants are denoted in red, rodents in yellow, bats in green, and swine in blue. Each viral and host specie used in the analysis is denoted in the left. (**B**) Topology comparison between the evolutionary history for the host (right) and the *Pestivirus* members (left). The numbers at the nodes indicate the divergence time for that node estimated using the BEAST software package. For better visualization, branches in the host and *Pestivirus* trees were colored following the coloring pattern used in panel A. Maximum clade credibility (MCC) trees based on *cyt b* sequences for the host and E2 gene for *Pestivirus* are presented. (**C**) Resolution of the *Pestivirus* phylogeny with their vertebrate hosts based on the methodology implemented in Jane. All possible codivergence, extinction, host-jumping, and lineage duplication events are shown following the Jane Manual [31]. To facilitate comprehension, the most relevant events linked to OVPV were denoted—cospeciation between *Ovine pestivirus* and sheep, cospeciation between TSV and sheep, and cospeciation between *Burdur pestivirus* and goat have been denoted with a line connected by two hollow colored circles. Host jumping events—from the common ancestor of TSV and *Ovine pestivirus* to classical swine fever virus are denoted using dashed arrows in gray color. Those species that supported cospeciation acting as donor species during just jump event are denoted, swine are denoted as a receptor species with the ability to accept new *Pestivirus* with further increasing in virulence.

To address the putative OVPV origin and its relationship with all potential hosts, a procrustean (PACo) analysis was performed. The PACo analysis produced a residual sum of squares (m2XY) of 0.289834, with an associated permutational *p* = 0.00014 (*p* < 0.001) based on 1 × 105 interaction, supporting, consequently, the overall congruence. The results obtained indicated that OVPV cospeciate with *Ovis aries* (Figure 7A). Likewise, other *Pestivirus* members showed a close relationship with their respective hosts including *Bat pestivirus* (BaPV) with *Myotis lucifugus*, *Burdur pestivirus* (BurPV) with *Capra hircus*, BDV, *Aydin pestivirus*, and TSV with *Ovis aries* as well as *Pronghorn pestivirus* with *Antilocapra Americana*, respectively.

The results showed that swine species are able to support the infection for six different pestivirus members including the well-known CSFV, *Bungowannah pestivirus*, BVDV1, and BVDV3 as well as the emergent viruses APPV and OVPV. This host species seems to acquire all these *Pestivirus* agents from host-jumping events, since none of the relationships among swine and pestiviruses were statistically supported, rejecting all the potential coevolutionary hypotheses.

For OVPV, the analysis determined that this virus is one the most recent emerging members of the *Pestivirus* genus, diversified from TSV, the common ancestor with CSFV, around 300 years ago (Figure 7B). The reconciliation of the *Pestivirus* tree with the host tree agreed with both previous findings revealed by PACo analysis and temporal reconstruction. The overall reconciliation comprised two cospeciations, eight duplications, four host switches, and 42 extinctions (including losses and failures to diverge) to a total cost of 60. The results evidenced that the expansion of *Pestivirus* genus has as a root in BaPV which cospeciated with its host and by subsequent host jumping and duplication events contributed to the diversification of the genus (Figure 7C). The OVPV and BurPV, infecting *Ovis aries* and *Capreolus capreolus*, respectively, seemed to emerge by a coevolution process after several duplication events from the ancestor BaPV. Other viruses, like TSV, also seem to coevolve in sheep (*Ovis aries*), after a duplication of the ancestor of OVPV and *Aydin pestivirus* resulted from a host jumping event of BurPV from these two closely-related host goat and sheep (Figure 7C).

## 4. Discussion

Currently, the demand for livestock products is rapidly increasing, reaching 33% of the agricultural gross domestic product (GDP) [32]. This has led to intensification of production, providing a hotbed for the spread and emergence of new diseases like porcine reproductive and respiratory syndrome in 1990s and swine influenza [33]. In this regard, a growing number of new pestiviruses have been discovered over the last few years, more recently APPV and LINDA, which have been associated with the occurrence of congenital tremor in pigs [3,4,5,6]. Interestingly, in 2017, a novel OVPV was isolated from aborted lamb fetuses in one farm in Italy. This virus showed high genomic identity with CSFV, while being in a separate phylogenetic cluster [12]. Considering the high homology of OVPV and CSFV, the pathogenic role of this virus needed to be elucidated.

The present study demonstrates the role of OVPV as a pathogenic agent causing trans-placental transmission in all the pregnant ewes infected at 68 dg. Remarkably, the high replication rate of OVPV detected in fetuses led to over 80% of prenatal deaths (abortion and stillbirths), and congenital persistent infection in the two lambs that were born. These animals showed severe weakness and other clinical signs, such as “hairy fleece”, which have been reported for BDV-persistently-infected lambs [34]. A lower proportion of trans-placental transmission and prenatal death was observed in experimental infections, carried out in pregnant ewes at a similar gestation time as the present study, with BDV genotype 4, BVDV-2, BVDV-1b, and *HoBi-like pestivirus* [19,20,35,36]. Therefore, the capacity of OVPV to induce prenatal death and congenital persistent infection in lambs was severe in comparison with previous experimental infections with other pestiviruses.

The high capacity to generate trans-placental transmission, as well as the replication rate detected in the OVPV-infected animals from the present study, suggest that this isolate may be of moderate virulence [37,38]. However, further studies will provide insight into the virulence of OVPV in lambs, taking into account that young animals are highly susceptible to *Pestivirus* infections [11,39].

It is worth highlighting that the cross-reaction in the CSFV-specific molecular diagnosis provides in vivo evidence for the close genomic relation between OVPV and CSFV. This is one of the most important findings from the present study, showing the diagnostic interference with CSFV [22], one of the most relevant diseases in swine. Depending on the epidemiological importance of OVPV, to be determined by further studies, as well as its possible ability to infect swine, new molecular diagnostic tests specific for CSFV need to be applied in the field. Due to the high identity between OVPV and CSFV in the 5′UTR, the CSFV-specific molecular diagnostic tests would need to avoid this region of the genome. Hence, in the present study the design and validation of the specific RT-qPCR assay for the detection of OVPV and its differentiation from CSFV was carried out.

Interestingly, OVPV infection induced high and early antibody response against the CSFV E2-glycoprotein, the most immunogenic CSFV protein. It should be noted that antibody response against CSFV has never been reported thus far in a species different from swine, the natural host of CSFV. Humoral response against CSFV in swine starts between the second and third weeks post-infection [11,40,41], whereas it was detected from the first week after OVPV infection in the ewes from the present study.

High titers of neutralizing antibodies were also detected in the ewes starting at the second week after OVPV infection, likely responsible for the short viremia and virus shedding period in these animals. However, despite the strong antibody response induced in the ewes, the virus persisted in tissues like tonsil and placenta for several weeks after infection, similar to infection with other pestiviruses, such as CSFV [41,42]. This may favor the prevalence of the virus in the field for long periods of time. Previous studies have shown that high neutralizing antibody response needs to be present at the moment of infection in order to avoid *Pestivirus* trans-placental transmission [43,44]. Accordingly, it is likely that OVPV fetal infection took place before the onset of the neutralizing antibody response.

The neutralizing antibody response detected in the OVPV-infected ewes was found to cross-react with all the *Pestivirus* strains analyzed, particularly against CSFV genotype 2 strains (Diepholz and Catalonia01). This is in agreement with a recent study carried out with field serum samples from sheep in which abortion had been related with OVPV [45]. However, the infection status of these ewes prior to OVPV infection was not known and previous infection with other pestiviruses could not be discarded. The high cross-reactivity found between OVPV and CSFV genotype 2 Diepholz strain is further explained by the high identity between these two viruses in the B/C domain of the E2-glycoprotein. This is relevant considering that this domain contains non-conserved epitopes, responsible for antigen specificity among various CSFV strains [27,46]. Other mechanisms involved in virus–host interaction may also be involved in the generation of the neutralizing antibody response generated by these viruses in different species. Further studies will clarify these findings.

Unravelling the mechanisms that determine the viral–host evolutionary process is essential to uncovering the nature and the determinants of emerging viral diseases [47]. To study more in-depth the mechanisms likely responsible for the high relationship found between OVPV and CSFV in the present study, the virus–host co-evolutionary process was studied in different viruses from the *Pestivirus* genus. Indeed, deciphering the evolutionary processes that allow novel pathogens to adapt to new hosts represents a major challenge for both public and animal health. It is unclear how the genetics and ecology of the viruses interact to shape the likelihood of successful cross-species transmission. In this regard, it has been suggested that population bottlenecks at inter-host transmission act as a major barrier to host adaptation, which limits the number of adaptive mutations that are able to cross the species barrier [48]. However, in the *Pestivirus* genus has been already indicated that a host-jumping event among relatively-distant hosts could lead to the emergence of new agents as CSFV with devastating consequences [27]. Notably, the emergence of CSFV was suggested through mathematical models to have taken place around the beginning of the 19th century, coinciding with an import of Tunisian sheep to the US [49,50]. In this context, it is likely that the intensive pig breeding practices, which were beginning to be employed, in combination with the housing of multiple animal species in the same farm (a common practice at the time), were responsible for the emergence of CSFV [27].

In agreement with this previous report, the phylodynamic analysis performed in the present study showed that CSFV, affecting pigs (*Sus scrofa*) seems to have emerged as the result from the inter-species jump of TSV from sheep (*Ovis aries*) to pigs (*Sus scrofa*) (Figure 7C). Notably, these analyses also showed that OVPV and CSFV share the TSV as a common ancestor and appeared to have emerged at around the same time (Figure 7C). This suggests that the differentiation of TSV into two new viruses (CSFV and OVPV), was likely favored by the close housing of multiple species (swine and sheep). Therefore, the human intervention for intensive livestock production likely favored not only the emergence of CSFV, but also OVPV.

The findings of the present study, regarding the possible emergence of OVPV more than 200 years ago raises the question of why this virus had not been detected thus far. The higher cross-reactivity in neutralizing antibody response against BDV than against CSFV genotype 1 strains (Alfort/187 and Margarita), which is routinely used in cross-reactivity neutralization panels, could have led to misdiagnosis of OVPV cases. Furthermore, the CSFV anti-E2 antibody ELISA test, proved to be more efficient for the detection of the humoral response induced by OVPV in the ewes. This test had not been previously used for analysis of sheep sera. It should be noted that a large number of ovine abortions are left without a final diagnosis in the field every year. It is possible that some of them may be due to infections caused by OVPV [51,52]. Interestingly, in the 1990s, one *Pestivirus* sequence isolated from a sheep showed high identity with CSFV and OVPV [12,53].

On the other hand, it was found that the antigenic reactivity between CSFV and TSV was the same as that between TSV and OVPV (69%). The similar antigenic reactivity found between OVPV and CSFV with TSV, as well as the high cross-reactivity in molecular diagnosis between OVPV and CSFV, may support the common ancestry found in the co-phylogenetic analysis. The results showed that swine could be a receptor species with the ability to accept new pestiviruses with further capability to increase virulence. Since the common ancestor of TSV and OVPV seems to be the ancestor for the emergence of CSFV in pigs, it is highly probable that OVPV can easily cross the host barrier infecting pigs with a further adaptation to this new host. Further studies are needed in order to clarify the possible role of OVPV in swine. It is worth mentioning that majority of viruses from the *Pestivirus* genus have been shown to cross the species barrier, for example, BVDV-1, BVDV-2, and BDV can infect multiple ruminant species and even wild ruminants, as well as some non-ruminants, like pigs [9,10].

Taken together, the present study showed the most likely origin and evolution for the emergence of OVPV as a new member of the *Pestivirus* genus, as well as its role as a pathogenic agent causing severe reproductive failures and congenital persistent infection in sheep. These results raise major concerns regarding the evolution, emergence, and barrier-crossing capacity of pestiviruses, suggesting the negative impact of human intervention and intensive animal production practices for the emergence of pathogens affecting multiple species, like CSFV or OVPV.

## Figures and Tables

**Table 1:** Viruses used in the validation of ovine pestivirus real-time TaqMan assay.

*Pestivirus* Species and Subgenotype	Reference Strain /Isolate	Source
BDV	BDV 137/4	Central Veterinary Laboratory (CVL), Weybridge, UK
	BDV FRITJNERS	Institute of Animal Science and Health (ID-DLO), Lelystad Netherlands
	BDV MOREDUM	EU Reference Laboratory for CSF, Germany
BVDV	BVDV NADL	EU Reference Laboratory for CSF, Germany
CSFV 1.1	Alfort 187	EU Reference Laboratory for CSF, Germany
	HCLV vaccine (C-Strain) [24]	CReSA, Spain
	Thiverval	CReSA, Spain
	Koslov	EU Reference Laboratory for CSF, Germany
	Glentorf	EU Reference Laboratory for CSF, Germany
CSFV 1.2	Brescia	Institute of Animal Science and health (ID-DLO) Lelystad Netherlands
	Baker	Central Veterinary Laboratory (CVL), Weybridge, UK
CSFV 1.4	Margarita	CReSA, Spain
	Pinar del Rio [25]	CReSA, Spain
CSFV 2.1	Paderborn (CSF277 reference strain)	CReSA, Spain
	Spain 97	Laboratori Sanitat Ramadera (Barcelona) Spain
CSFV 2.2	CSF573 reference strain (Italy Parna’98)	CReSA, Spain
CSFV 2.3	Diepholz (CSF104 reference strain)	EU Reference Laboratory for CSF, Germany
	Catalonia 01 [18]	CReSA, Spain
	Spreda (CSF123 reference strain)	CReSA, Spain
	Rostock (CSF184 reference strain)	EU Reference Laboratory for CSF, Germany
	Uelzen (CSF639 reference strain)	CReSA, Spain
APPV	Nasal swab from field-infected pig	CReSA, Spain
	Thymus from field-infected pig	CReSA, Spain
Novel ovine pestivirus (OVPV)	Inoculum Italian ovine pestivirus [12]	Istituto Zooprofilattico Sperimentale della Lombardia e Dell’Emilia Romagna-IZSLER

**Table 2:** Neutralizing antibody titers against homologous and non-homologous viruses from the infected ewes.

Animal Number	6 dpi	13 dpi	20 dpi	27 dpi	34 dpi	41 dpi							
	Homologous Virus (Italy OVPV)						CSFV Alfort/ 187	CSFV Margarita	CSFV Diepholz1	CSFV Catalonia01	BVDV NADL	BDV 137/4	BDV-pig-SP-2007
Ewe 1	Neg. ^a^	1/160	1/640	1/320	1/5120	1/10240	1/80	1/40	1/1280	1/1280	1/40	1/80	1/320
Ewe 2	Neg.	1/320	1/1280	1/1280	1/1280	1/5120	1/80	1/60	1/2560	1/1920	1/160	1/320	1/160
Ewe 3	Neg.	1/80	1/160	1/1280	1/1280	1/5120	1/80	1/30	1/920	1/3840	1/80	1/20	1/320
Ewe 4	Neg.	1/640	1/1280	1/1280	1/640	1/2560	1/20	1/20	1/15360	1/2560	1/20	1/40	1/160
Ewe 5	Neg.	1/320	1/1280	1/1280	1/1280	1/1280	1/10	1/15	1/640	1/320	1/10	1/40	1/40
Ewe 6	Neg.	1/320	1/2560	1/640	1/1280	1/5120	1/20	1/30	1/3840	1/960	1/40	1/160	1/160
Ewe 7	Neg.	1/160	1/640	1/2560	1/5120	1/5120	1/40	1/160	1/30720	1/3840	1/80	1/640	1/160
Ewe 8	Neg.	1/320	1/1280	1/2560	1/2560	1/1280	1/20	1/60	1/5120	1/960	1/80	1/40	1/160
Mean value	**/**	**1/290**	**1/1140**	**1/1400**	**1/2320**	**1/4480**	**1/44**	**1/51**	**1/7555**	**1/1960**	**1/64**	**1/168**	**1/185**
SD	**/**	**171**	**713**	**801**	**1808**	**2903**	**31**	**46**	**10511**	**1340**	**48**	**215**	**93**

^a^ Neg. = Negative.

## Supplementary Materials

The following are available online at https://www.mdpi.com/1999-4915/12/7/775/s1, Table S1: Sequence information of *Pestivirus* strains used in the current study. Table S2: Sequence information of *Pestivirus* host species used in the current study.
